# A comparative assessment of mosquito trapping methods for vector surveillance in Scotland

**DOI:** 10.1186/s13071-026-07478-y

**Published:** 2026-06-05

**Authors:** Meshach Lee, Georgia Kirby, Rebecca E. Brown, Alanna McCallum, Jean-Philippe Parvy, Susanne Krabbendam, Emilie Pondeville, Jolyon M. Medlock, Alexander G. C. Vaux, Luca Nelli, Francesco Baldini, Heather M. Ferguson

**Affiliations:** 1 School of Biodiversity, One Health and Veterinary Medicine, University of Glasgow, Glasgow, UK; 2 MRC-University of Glasgow Centre for Virus Research, Glasgow, UK; 3 Medical Entomology and Zoonoses Ecology, UK Health Security Agency, Porton Down, UK

**Keywords:** Mosquitoes, Trap comparison, Vector surveillance, CDC light trap, Mosquito Magnet, BG pro trap, BG Sentinel, Scotland, *Anopheles claviger*

## Abstract

**Background:**

While mosquito vector-borne disease (VBD) risk remains low in Northern Europe, surveillance is needed to monitor potential vector species. However, most mosquito trapping methods have been evaluated only in areas of high mosquito density, with limited knowledge of their sensitivity in low-abundance areas. Here, we assessed the relative performance of four commercially available traps in an urban setting in Scotland, where mosquito density is low.

**Methods:**

Two trap evaluation experiments were conducted in an urban marsh in Glasgow City during August 2023. In experiment 1, the Biogents BG-Pro (BGP), Biogents BG-Sentinel (BGS), and CDC miniature light traps (LT) were compared over 15 days using a 3 × 3 Latin square design. A subsequent experiment incorporated the Mosquito Magnet (MM) trap to compare it with the other three traps over 12 days using a 4 × 4 Latin square design. Alongside these experiments, two CDC gravid traps (GT) were trialled across 24 days to target blood-fed and gravid mosquitoes. Mosquitoes were morphologically identified to species and species-group level, and polymerase chain reaction (PCR) assays were used to identify *Culex pipiens* s.l. biotypes.

**Results:**

Over the 96 trapping events, 150 mosquitoes were collected from eight species. Mosquito abundance was generally low (mean abundance: 1.61 per day ± 1.89). The most abundant species collected was *Anopheles claviger* (*n* = 63), followed by *Culiseta morsitans* (*n* = 28). In Experiment 1, there was no significant difference in mean mosquito abundance among the BGS, BGP, and the LT. In Experiment 2, there was a significant positive association between the MM and daily mosquito abundance. The MM also collected more species than the other trap types (6 compared to 1–4 species). Gravid traps collected 19 mosquitoes representing three species, of which 21% were gravid or blood-fed.

**Conclusions:**

While the MM caught more mosquitoes than other methods, this was likely due to its more consistent and higher-volume CO_2_ output. However, important potential vector groups (*Culex*) were present only in the BGS, BGP, and LT. No single method consistently captured all species, indicating that a combination of methods may be necessary for comprehensive vector surveillance in Scotland.

**Graphical Abstract:**

**Figure:**
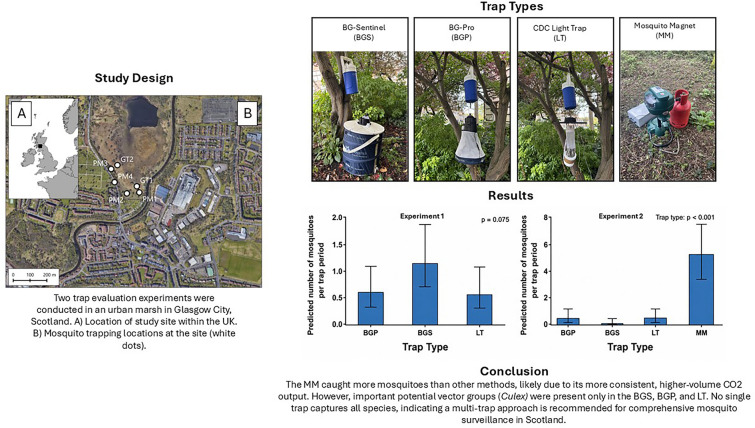

**Supplementary Information:**

The online version contains supplementary material available at 10.1186/s13071-026-07478-y.

## Background

Mosquitoes are a significant public and veterinary health threat worldwide, transmitting several important vector-borne diseases (VBDs) [1]. Vector surveillance is essential for managing endemic and emerging VBDs [2, 3], especially in new geographic areas or host populations [4]. This is particularly crucial in the context of climate and other associated environmental changes, which are expected to expand the distribution of many mosquito-borne VBDs into more northern latitudes [5–7].

Environmental change is increasing the risk of mosquito-borne VBDs in the UK [8], exemplified by the recent confirmation of circulating Usutu virus (USUV) in birds and mosquitoes in England and the first detection of West Nile Virus (WNV) in mosquitoes in England [9, 10]. Assessing these risks and the potential transmission dynamics of mosquito VBDs in the UK requires a detailed understanding of local vector ecology. At least 36 mosquito species have been recorded in the UK [11], with several having potential veterinary and public health importance [7, 12–16]. Amongst native mosquito species, the *Culex pipiens* s.l. species complex poses the most imminent risk for VBD transmission in the UK. This complex comprises two biotypes: *Cx. pipiens pipiens*, which primarily feeds on birds, and *Cx. pipiens molestus*, which feeds on both birds and other mammals, such as humans [7, 12]. Together, these species are among the primary vectors of several zoonotic VBDs emerging in Europe, including USUV and WNV [17]. Effective trapping methods for all these species are therefore needed to assess the current status of vector populations and future VBD risk in the UK. While there has been extensive surveillance of mosquito populations in England and Wales [18–22], until recently, there has been limited investigation of mosquito population distribution in Scotland [23, 24]. Thus, robust data on the distribution, abundance, and demography of potential vector species in Scotland are essential. A recent large-scale survey of mosquitoes in Scottish wetlands provided a valuable first step for this [23], highlighting that mosquitoes are widespread but occur at relatively low density in Scotland and confirming several potential vector species, including the *Cx. pipiens molestus* biotype. However, additional surveillance, particularly in urban areas, is necessary to give a fuller understanding of human exposure to mosquitoes.

Currently, 23 mosquito species have been reported in Scotland [23, 24]. However, the relatively low mosquito densities in Scotland may make surveillance of these populations more difficult, requiring approaches that are highly sensitive and capable of detecting a range of species. In particular, trapping methods must be capable of sampling key vector species of interest, including the different biotypes of *Culex pipiens* s.l. species complex. A range of commercially available mosquito traps have been used for mosquito surveillance in both tropical [25–28] and European settings [29–31], each exploiting different mosquito behaviours, e.g. host-seeking, oviposition, or resting. These approaches are often biased towards specific life-history traits associated with these behaviours, so selecting the correct method is important for answering specific surveillance questions. Host-seeking traps are often the most widely used, focusing on collecting female mosquitoes seeking a blood meal [32], and they generally yield higher numbers of females than those that target other life-history stages [33–35]. In contrast, they are often biased towards newly emerged mosquitoes that have not yet had their first blood meal, thereby underestimating the age structure and the proportion of the population infected with blood-borne pathogens [32]. Conversely, gravid/oviposition traps focus on collecting mosquitoes as they attempt to oviposit, typically after they have taken a blood meal, increasing the probability of sampling an infected mosquito [36]. These traps, however, are often specifically designed to exploit the egg-laying behaviour of container-breeding species, which limits their application for the surveillance of a broader range of species [36, 37]. These differences in the efficacy of trapping methods highlight the importance of understanding which methods are suitable for surveillance, particularly in understudied regions.

Amongst mosquito host-seeking traps, the CDC miniature light trap [38] (LT; using a light source and often paired with a CO_2_ bait) is widely used to trap malaria vectors in Africa (*Anopheles* species) [39, 40]. Other traps made by Biogents [41], the BG-Sentinel (BGS) or BG-Pro (BGP) traps, are often used to collect *Aedes* (*Stegomyia*) spp. vectors globally [42, 43]. These traps primarily use olfactory lures and CO_2_. All of these methods are relatively easy to deploy in the field as they are light, portable, and can be powered by 6- or 12-V batteries. The Mosquito Magnet (MM) [44] was developed as a household outdoor mosquito trap to reduce human biting [45, 46]. Compared with the other traps, this is a relatively large, less portable device that requires a propane tank to produce CO_2_ lure and can be combined with an olfactory lure. This approach has also been successfully used for mosquito surveillance in the UK [20, 22] and globally [47–49].

Despite the availability of various trap types, selecting the most effective sampling method for a given context is challenging because host-seeking traps often vary in efficacy across settings [50–59]. In the UK, formal comparative evaluations of mosquito trap performance remain limited. However, a few studies in southeast England showed that the MM had considerably more species and a larger number of mosquitoes than both the BGS and the LT [60, 61]. However, the MM struggled to collect the key vector species for USUV and WNV, *Culex pipiens* s.l., in this setting. This highlights that trap choice is highly context- and target species-dependent, emphasising the importance of formal comparisons, particularly in new ecological contexts such as Scotland.

Gravid traps may also have value as a mosquito surveillance tool in Scotland, given their demonstrated success in collecting mosquitoes elsewhere in the UK [62, 63]. These traps are already incorporated into the UK's surveillance plan for invasive mosquitoes [64] and have proven successful in collecting *Culex* and *Culiseta annulata* at both urban and rural sites in England [62]. Their demonstrated effectiveness in the UK highlights the potential value of gravid traps as a supplementary sampling tool; however, they have never been used for mosquito surveys in Scotland except for port surveillance [65].

A comprehensive assessment of mosquito VBD establishment in areas such as Scotland, where diverse vector groups may be present but at low densities, will require efficient vector surveillance approaches. This study assessed the performance of four widely used trapping methods for sampling mosquito populations at an urban wetland in Scotland: MM, BGS, BGP, and LT. The primary aim was to compare the efficacies of these methods in terms of the number and diversity of mosquitoes collected. In addition, we conducted a preliminary study to evaluate the performance of CDC gravid traps for mosquito collection at our study site. Unlike the other trapping methods, which were formally evaluated and compared to one another in a Latin square experiment, the gravid traps were used only to demonstrate proof of principle that this method could sample mosquitoes in our setting. To our knowledge, there are no previous reports of the use of this method in Scotland. These results will help to inform future trapping protocols to enhance ongoing mosquito surveillance in Scotland.

## Methods

### Study area

**Fig. 1:**
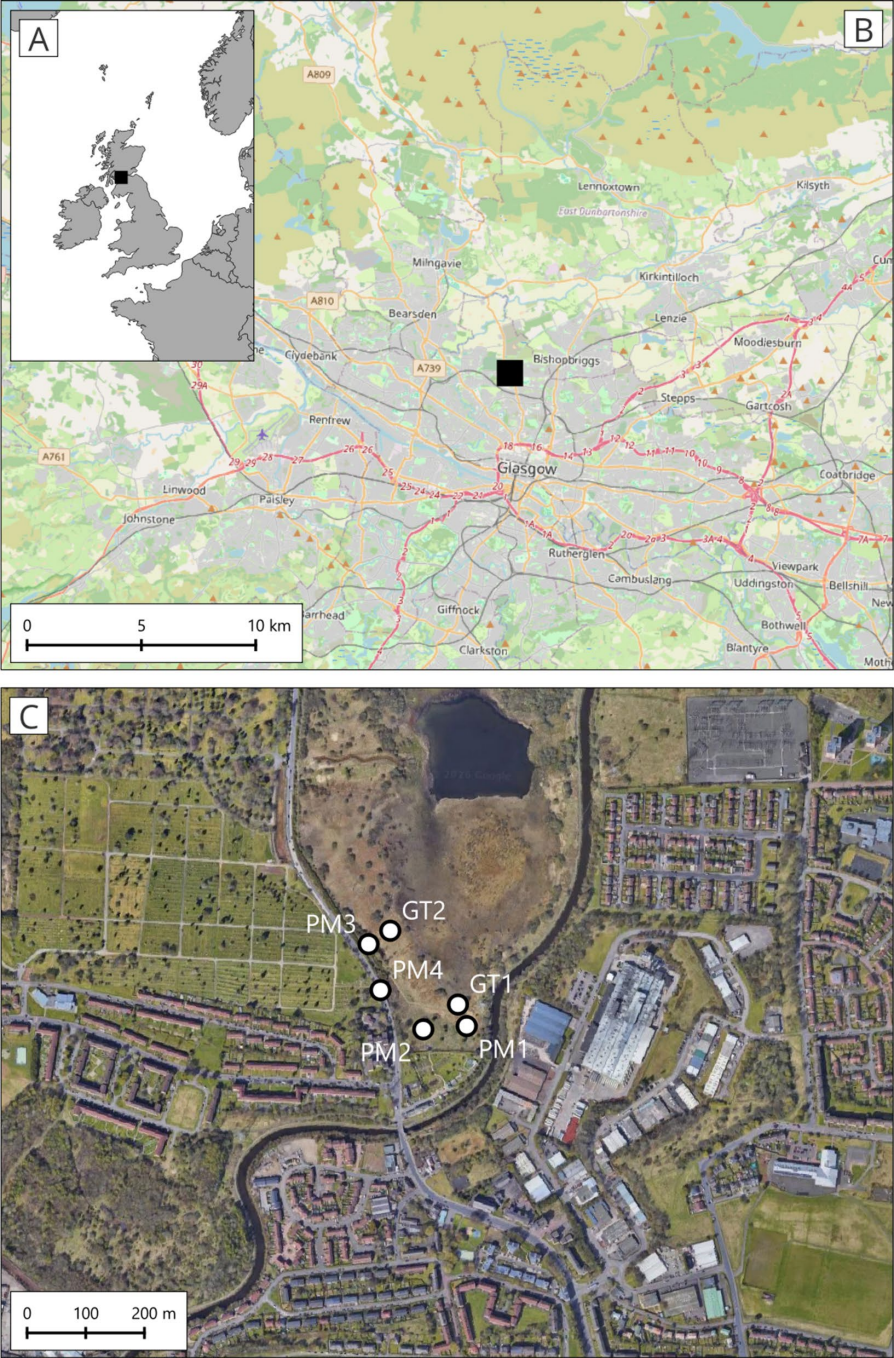
Study locations and mosquito trapping sites at Possil Marsh, Glasgow, Scotland. **A** Location of Possil Marsh within the UK. **B** Location of Possil Marsh within the city of Glasgow. **C** Mosquito trapping locations (white dots). PM1–PM4 (Possil Marsh trapping locations 1–4) refer to the host-seeking trap locations used in the Latin square design for Experiments 1 and 2, while GT1 and GT2 indicate fixed locations of the CDC gravid traps

This study was conducted at Possil Marsh (55.546859°N, 4.1554876°W), an urban wetland nature reserve in Glasgow managed by the Scottish Wildlife Trust, consisting of two main habitat types: reedbeds and wet woodland (Fig. 1A).

### Study Design

This study consisted of two experiments. Experiment 1 compared the BGS, BGP, and LT using a 3 × 3 Latin square design over 15 trapping days (5 rounds, 7 August 2023–21 August 2023). The three trapping locations in Possil Marsh (PM 1–3, Fig. 1B) were positioned at least 75 m apart to limit competition between traps. This minimum distance was selected based on the consensus that host-seeking traps do not compete with one another on this scale, as reflected by their common use in previous studies [34, 61, 66]. Experiment 2 compared the BGS, BGP, MM, and LT using a 4 × 4 Latin square design over 12 days (3 rounds, 22/08/2023—07/09/2023). This experiment was conducted at the same three trapping stations as in Experiment 1 (PM 1–3), with an additional station added (PM4, Fig. 1B).

On each day of the experiments, one trap of each type was assigned to one trapping station, with allocated traps being turned on at approximately the same time every morning and run for 24 h (to allow the collection of diurnal and nocturnal species). After 24 h, the collection bags were removed, and the traps were rotated consecutively to a different sampling station before being returned to the original sampling station. This rotation aimed to limit the effect of site-specific variation by ensuring each trap type was used at each sampling station in a single round. Thus, a complete sampling round lasted 3 consecutive days for Experiment 1 and 4 for Experiment 2. The catch bags/collection cups were immediately placed in a portable freezer for sample preservation. Trap batteries were replaced every 24 h to ensure continuous operation.

Alongside the two Latin square experiments, we trialled two CDC gravid traps (GTs) to assess their capacity to trap mosquitoes in Scotland. These traps ran for 24 days (9 August 2023–6 September 2023). This trap type was not incorporated into Latin squares and was run only at fixed sites. The gravid traps were placed 25 m apart from two sampling stations, PM1 and PM3, and were labelled GT1 and GT2 (Fig. 1B). Collection bags and batteries were replaced every 48 h, with 24 collections made over the sampling period.

### Trapping methods

The BGS (SI Figure S1A) (BioGents, Regensburg, Germany, [41]) was placed at ground level in a shaded area and was baited with a combination of carbon dioxide and a BG lure™ (an olfactory lure containing lactic acid, ammonia, and caproic acid, which mimic human skin odour cues; BioGents, Regensburg, Germany), with the trap powered by a 12-V battery. The BGP (BioGents, Regensburg, Germany) was hung 1.5 m above ground level and baited with CO_2_ and a BG lure™ (SI Figure S1B); it was powered by a 6-V battery. The LT (SI Figure S1C, John W. Hock, [38]) was also hung 1.5 m above the ground and baited with CO_2_ and light to attract insects; it was also powered by a 6-V battery. The CO_2_ for these traps was produced by mixing 8 g of dried yeast with 80 g of sugar in 800 ml of warm water (about 45°C, the optimum temperature for yeast activation [67]). This mixture was then placed in an Insulated Dry-Ice Container (Igloo) (John W. Hock, [38]), designed to buffer against ambient temperature fluctuations. The open emission point of the Dry-Ice Container was then placed directly above the trap (SI Figure S1), and the mixture was replaced daily. A yeast fermentation method was selected because of logistical challenges that prevented the use of other methods, including an inconsistent supply of dry ice and lack of access to CO_2_-vapour withdrawal bottles due to a national shortage during the study year. This fermentation method typically produces 3–5 ml/min of CO_2_ under laboratory conditions of 20–25 °C [68, 69]. The Mosquito Magnet (MM) (MosquitoMagnet, Lititz, PA, USA; [44]) used in this study was a Patriot Plus MM4202B, modified to run from a 12-V car battery rather than mains power (SI Figure S1D). This trap was baited with a Mosquito Magnet® Octenol Attractant (an olfactory lure containing octenol, a chemical found in the breath of large mammals; MosquitoMagnet, Lititz, PA, USA), and approximately 462.96 ml/min of CO_2_ was produced by the trap through the conversion of propane gas [61]. The CDC gravid trap (GT, SI Figure S1E, John W. Hock, [38]) consists of a collection bag and a pan containing an oviposition medium. The medium consisted of 0.5 kg hay and 5 l water; this medium was left to infuse for 7 days before the experiment began.

Environmental data were collected at two spatial scales to characterise conditions potentially influencing mosquito catches: microclimatic conditions at each trapping station and site-level daily weather for the wider study area. Microclimatic conditions were recorded at each sampling station on each trapping day using a Tinytag data logger (TGP-4017; Gemini Data Loggers, UK [70]). For each 24-h trapping period, the daily mean, minimum, and maximum summaries of air temperature (TT mean temp, TT min temp, and TT max temp; °C) and relative humidity (TT mean RH; %) were derived. To capture broader day-to-day meteorological variation, site-level weather from the nearest Met Office station (Mugdock Country Park; 55.973, − 4.329; ~ 9 km from Possil Marsh). These data comprised daily mean, minimum, and maximum temperature (WS mean temp, WS min temp and WS max temp; °C) and total daily rainfall (WS total rainfall; mm). All environmental variables were aligned to the corresponding 24-h trapping periods for subsequent analyses.

### Mosquito identification and processing

After removal from the trap, catch bags/collection cups were kept at − 20 °C to kill mosquitoes. Morphological identification to the species/species group level was conducted using a key [71]. The sex, species/species group, and physiological status (unfed, blood-fed at collection, or gravid) of each mosquito sample were recorded based on visual inspection [72].

### Molecular identification

Further molecular identification by polymerase chain reaction (PCR) was performed on all female *Culex* mosquitoes to confirm whether they were *Culex torrentium* or *Cx. pipiens* s.l., and for the latter*,* whether they were the *Cx. pipiens* or *Cx. molestus* biotype. This was achieved by extracting *Culex* DNA from either a single leg or, in cases where no legs were present, the head of the mosquito. These were then manually ground using proteinase K (200 µg/ml) and 20 µl of squishing buffer (10 mM Tris–HCl, 1 mM EDTA, 25 mM NaCl) and incubated at 37 °C for 20 min. [73]. These samples were then inactivated by incubation at 95 °C for 2 min.

The first PCR to identify *Culex torrentium* or *Cx. pipiens* s.l. was performed using the ACEpipF, ACEtorrF, and B1246sR primers [74], and the second PCR was used to identify the *Cx. pipiens* s.l. sample biotype was performed using the molCQ11R, pipCQ11R, and CQ11F2 primers [75]. Both PCRs were conducted under the following conditions: an initial denaturation at 95 °C for 3 min, followed by 40 cycles at 95 °C for 30 s, 54 °C for 30 s, and 72 °C for 20 s, with a final extension at 72 °C for 10 min. The amplified fragments were then visualised using a 2% agarose gel. Full details are available in SI Text S1. If there was no amplification after either PCR assay, the sample was marked as inconclusive. Molecular identification of these species was prioritised, given their primary role as vectors of emerging VBDs in Europe and the UK (USUV and WNV) and the different epidemiological implications of the two *Cx. pipiens* s.l. biotypes.

### Statistical analysis

All mosquito count data were analysed in R version 4.4.2. Generalised linear mixed models (GLMMs) were fitted using the package ‘glmmTMB’ [76] to assess the effect of trap type and environmental variables on the total number of mosquitoes caught per trapping period (all species combined). Analyses were conducted on total mosquito abundance because the low sample sizes for individual species prevented reliable species-specific model fitting.

For Experiment 1, two GLMMs were fitted. The first model examined site-level environmental variables (WS min temp, WS max temp, and total WS rainfall) as explanatory variables, with mosquito abundance as the response. The second model focused on microclimatic conditions at trapping stations (TT mean temp and TT mean RH). In both models, trap type was included as a fixed effect, with site and date included as random effects. This structure enabled comparison between broader site-level weather influences and fine-scale microclimate effects on mosquito catches. A Poisson distribution was used because no overdispersion or zero-inflation was detected (tested using the ‘performance’ and ‘DHARMa’ packages) [77, 78].

Experiment 2 followed the same structure, with two GLMMs fitted: one using site-level weather station data (WS min temp, WS max temp, and WS total rainfall), and another using microclimate data from dataloggers (TT min temp, TT max temp and TT mean RH). Trap type was included as a fixed effect in both models, with site and date as random effects. For Experiment 2, GLMM 1 used a Poisson distribution; however, due to overdispersion, GLMM 2 used a negative binomial distribution.

Temperature variables (mean, minimum, and maximum) were highly collinear with both site-level and microclimate variables; therefore, to address this collinearity, two full candidate models were specified for each analysis. For the site-level data, one model included WS mean temp, while the other included WS min temp and WS max temp. Both models also included rainfall and trap type as fixed effects, with site and date as random effects. For microclimate data, one model included TT mean temp, while the other included TT min temp and TT max temp. Both models also included mean TT, mean RH, and trap type as fixed effects, with site and date as random effects.

Competing model structures were compared using Akaike’s information criterion (AIC) using the ‘AICcmodavg’ package [79], and the model with the lower AIC was retained for subsequent model selection. Backwards stepwise elimination based on likelihood ratio tests was then used to assess the contribution of explanatory variables in the retained final model, using the ‘stats’ package, and model assumptions were checked using ‘DHARMa’ [78]. In models where trap type was significant, pairwise comparisons were conducted using ‘emmeans’ [80] to assess differences between trap types. Model predictions were extracted using the ‘ggeffects’ package [81] and plotted using ‘ggplot2’ [82].

Mosquito diversity by trap types was compared using species richness (total number of species or species groups per trap), the Gini-Simpsons index [83], and the Shannon index [84]. The Gini-Simpson index represents the probability that two randomly sampled individuals belong to the same species and gives greater weight to the dominant species. Shannon's index quantifies uncertainty in predicting the species identity of a randomly sampled individual and is more sensitive to rare species. Full details of the formulas used to calculate these diversity indexes, along with their confidence intervals, are given in SI Text S2.

## Results

One hundred fifty mosquitoes representing eight species were collected over 96 trapping events (7 August 2023–7 September 2023). The mean site-level temperature and rainfall were higher during Experiment 1 (14.99 °C ± 0.42/ 2.89 mm ± 0.9) than during Experiment 2 (13.78 °C  ± 0.5353/1.29 mm ± 0.47) (SI Table S1), a trend also observed in the microclimate data (SI Table S2). In both experiments, PM1 exhibited the highest and lowest temperatures, whilst PM2 demonstrated the highest relative humidity (SI Table S2). In Experiment 2, PM1 also showed the highest relative humidity (SI Table S2).

### Experiment 1

**Fig. 2:**
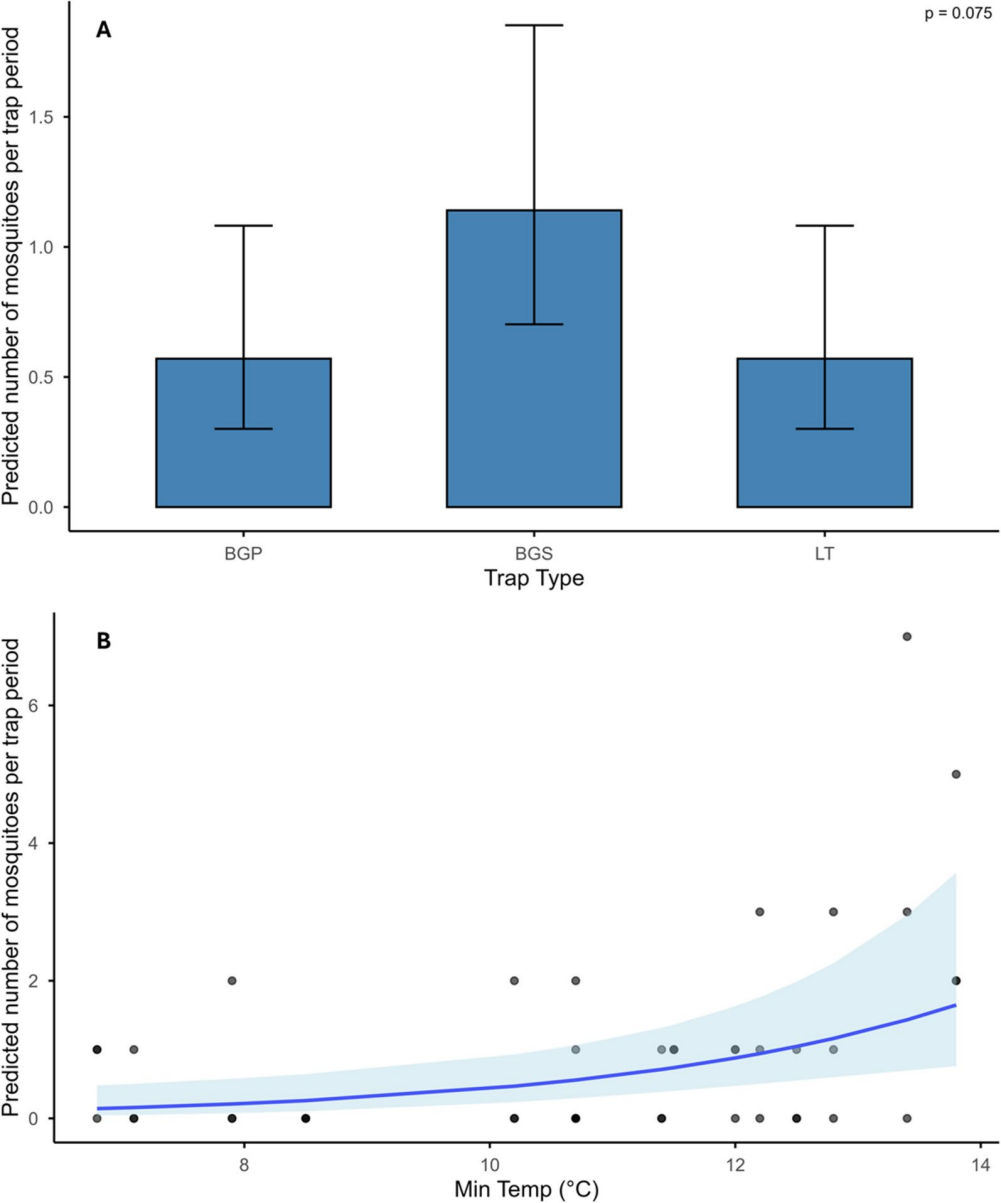
Modelled relationships from Experiment 1 at Possil Marsh, Glasgow. **A** The mean predicted number of mosquitoes caught per 24-h trapping period by the BGP, the BGS, and the LT from GLMM 1 (site-level environmental model). **B** Relationship between minimum daily site-level temperature (°C) and the mean predicted daily mosquito count. Error bars and shaded areas represent 95% confidence intervals

Forty-four mosquitoes belonging to five species were collected during Experiment 1. The most common species was *Culiseta morsitans* (47.7%), followed by *Culex pipiens* s.l. (27.3%, of which six were female and six were male; Table 1). PCR identification of the six *Cx pipiens* s.l. females confirmed five as *Cx. pipiens pipiens*, whilst one failed to amplify. The BGS trap caught the most mosquitoes, followed by the BGP and LT traps, which had similar counts (Table 1). However, although the total mosquito count varied somewhat between trap types, there was no statistically significant difference in the predicted mean abundance per day in either GLMM (SI Table S3, Fig. 2A). Of the environmental covariates considered, only the minimum site-level daily temperature (from GLMM 1, which included WS min temp and WS max temp site-level daily temperature SI Table S3 and S4) was significant, with higher minimum temperatures positively associated with mosquito abundance (Fig. 2B).

**Table 1:** The total number and percentage of each mosquito species collected per trap type and the mean abundance per 24-h trapping period (mean ± 95% confidence interval)

Species	BGP (%)	LT (%)	BGS (%)	MM (%)	Total
Experiment 1					
*Aedes cinereus/Ae. geminus*	3 (50)	0	3 (50)	NA	6 (13.64)
*Culex pipiens* s.l.	1 (8.33)	4 (33.33)	7 (58.33)	NA	12 (27.27)
*Culiseta annulata*	1 (25)	0	3 (75)	NA	4 (9.09)
*Cs. morsitans*	6 (28.57)	7 (33.33)	8 (38.10)	NA	21 (47.73)
*Coquillettidia richiardii*	0	0	1 (100)	NA	1 (2.27)
Total	11 (25)	11 (25)	22 (50)	NA	44
Mean abundance per trapping period	0.73 ± 0.54	0.73 ± 0.39	1.47 ± 1.01	NA	
Experiment 2					
*Aedes cantans*	0	1 (50)	0	1 (50)	2 (1.89)
*Aedes cinereus/Ae. geminus*	0	0	0	16 (100)	16 (15.09)
*Aedes punctor*	0	0	0	4 (100)	4 (3.77)
*Culex pipiens* s.l.	3 (50)	2 (33.33)	1 (16.67)	0	6 (5.66)
*Anopheles claviger*	0	0	0	63 (100)	63 (59.43)
*Cs. annulata*	1 (12.5)	2 (25)	0	5 (62.5)	8 (7.55)
*Cs. morsitans*	3 (42.86)	3 (42.86)	0	1 (14.29)	7 (6.60)
Total	7 (6.60)	8 (7.55)	1 (0.94)	90 (84.91)	106
Mean abundance per trapping period	0.58 ± 0.36	0.67 ± 0.48	0.08 ± 0.16	7.5 ± 4.36	

Experiment 1 ran over 15 days at Possil Marsh, Glasgow (7 August 2023–21 August 2023). Experiment 2 ran over 12 days at Possil Marsh, Glasgow (22 August 23–7 September 2023). Mean abundance was calculated per trap per 24-h sampling period

Across trap types, species richness and both Shannon and Gini-Simpson diversity indices were highest for the BGS, followed by the BGP and LT (Table 2).

**Table 2:** Mosquito species diversity measures (Species richness, Shannon's and Gini-Simpson indices) for the different trap types used in Experiments 1 and 2 at Possil Marsh, Glasgow

Experiment number	Trap type	Species richness	Shannon’s index	Gini-Simpson index (± 95% confidence interval)
1	BGP	4	1.121	0.612 ± 0.093
	LT	2	0.655	0.463 ± 0.095
	BGS	5	1.416	0.727 ± 0.041
2	BGP	3	1.004	0.612 ± 0.150
	LT	4	1.321	0.718 ± 0.120
	BGS	1	0	0
	MM	6	0.956	0.473 ± 0.011

Most mosquitoes were classified as unfed. Two gravid *Cs. morsitans,* one gravid *Cx. p. pipiens*, and one blood-fed *Culiseta annulata* were collected by the BGS, and one gravid *Cs. morsitans* by the BGP.

### Experiment 2

A total of 106 mosquitoes were collected in Experiment 2 (Table 1), representing seven species. Most mosquitoes were caught in the MM (n = 90). *Anopheles claviger* was the most abundant species (*n* = 63) and was collected only in the MM. In contrast to Experiment 1, *Cs. morsitans* (6.6%) and *Cx. pipiens* s.l. (5.7%, of which three were female and three were male) accounted for only a small proportion of the total mosquitoes collected. Of the three *Cx. pipiens* s.l. females caught, two were confirmed by PCR to be *Cx. p*. *pipiens*, whilst one failed to amplify.

**Fig. 3:**
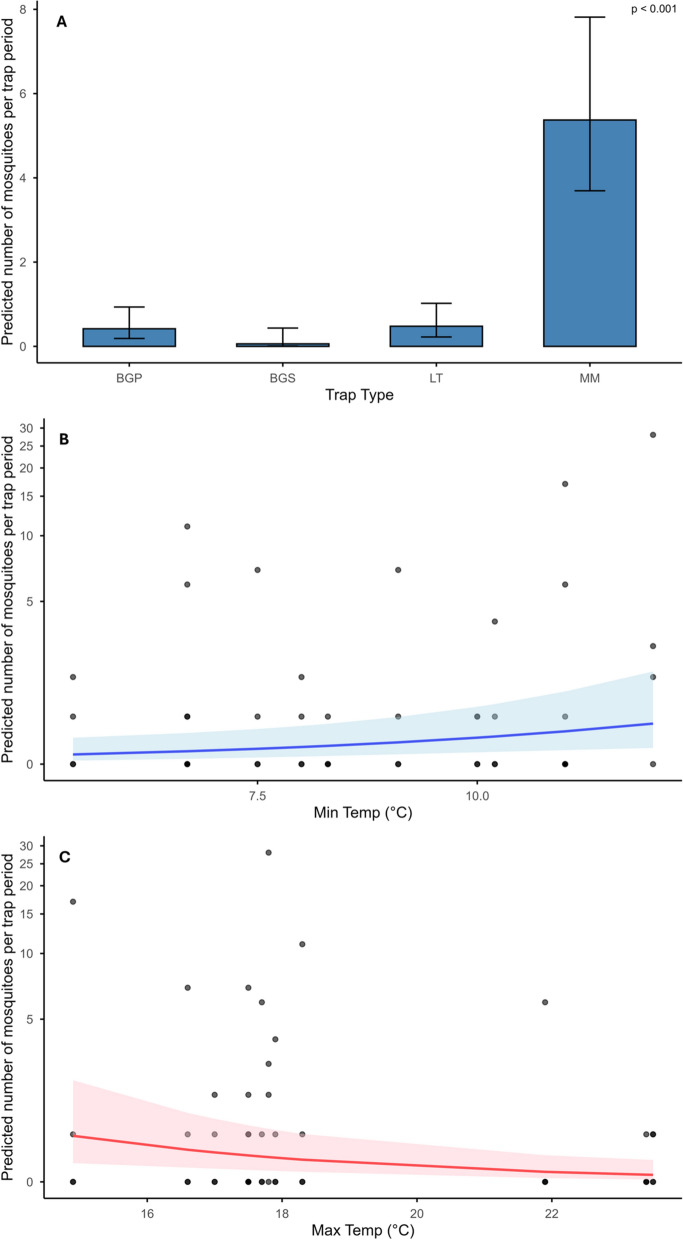
Modelled relationships from Experiment 2 at Possil Marsh, Glasgow. **A** The mean predicted number of mosquitoes caught per 24-h trapping period by the BG-Pro (BGP), the BG-Sentinel (BGS), the CDC light trap (LT), and the Mosquito Magnet (MM) from GLMM 1. **B** Relationship between minimum daily site-level temperature (°C) and the mean predicted daily total mosquito count. **C** Relationship between maximum daily site-level temperature (°C) and the mean predicted daily total mosquito count. Error bars and shaded areas represent 95% confidence intervals. To improve visualisation of low counts, the y-axis for plots **B** and **C** is shown on a square-root scale

Trap type was a significant predictor of mosquito abundance, when considering both site-level environmental conditions (GLMM1; X^2^ = 162.99, *P* < 0.001, Fig. 3A) and microclimatic variation at trap sites (GLMM2; X^2^ = 38.876, *P* < 0.001; SI Table S3). The MM collected significantly more mosquitoes than the BGS (GLMM1: estimate = − 4.500, SE = 1.010, *P* < 0.001; GLMM2: estimate = − 4.439, SE = 1.050, *P*  < 0.001), BGP (GLMM1: estimate = − 2.554, SE = 0.392, *P* < 0.001; GLMM2: estimate = − 2.508, SE = 0.489, *P* < 0.001), and LT (GLMM1: estimate = − 2.420, SE = 0.369, *P* < 0.001; GLMM2: estimate = − 2.389, SE = 0.477, *P* < 0.001) (SI Table S5). No significant differences in mosquito abundance were observed between the remaining three trapping methods in either model. At the site-level, daily maximum temperature was negatively associated with mosquito abundance (Fig. 3B; SI Table S3 and S4), and mosquito abundance was predicted to rise with minimum temperature (Fig. 3C; SI Table S3 and S4). None of the microclimatic variables measured at trapping sites (daily RH, minimum and maximum temp) were significantly related to mosquito abundance in Experiment 2 (SI Table S3).

Compared with the other trap types, the MM collected a higher number of all mosquito species, except for *Cx. pipiens* s.l. (which were notably absent in the MM) and *Cs. morsitans* (Table 1). The BGP collected the most *Cx. pipiens* s.l. in Experiment 2 and collected similar numbers of *Cs. morsitans* to the LT. The Shannon and Gini-Simpson indices indicate that mosquito diversity was highest in the LT, followed by the BGP, MM, and BGS (Table 2). However, the MM had the highest species richness, with six species. As in Experiment 1, most of the mosquitoes sampled were in an unfed state. Two gravid *Cx. p. pipiens* and one gravid *Cs. morsitans* were collected in the BGP, one gravid *Cx. p. pipiens* collected by the LT and one gravid *Anopheles claviger* collected by the MM.

### Gravid trap pilot study

Nineteen mosquitoes from three species were collected during the 24 days the gravid traps were in operation. Most were in the genus *Culiseta*, with equal numbers of *Cs. annulata* and *Cs. morsitans* (Table 3). Most mosquitoes caught during this experiment were unfed females, although two gravid *Cs. annulata*, two blood-fed *Cs. morsitans*, and two male mosquitoes were also caught.

**Table 3:** Summary of mosquito species caught in two CDC gravid traps (GT) run over 24 days at Possil Marsh, Glasgow (9 August–6 September 2023)

Species	GT1	%	GT2	%	Total
*Aedes cinereus/Ae. geminus*	0	0	1	8.33	1
*Culiseta annulata*	3	42.8	6	50	9
*Cs. morsitans*	4	57.14	5	41.67	9
Total	7		12		19

## Discussion

This study, to our knowledge, is the first assessment of the efficacy of the Biogents Sentinel trap (BGS), the Biogents-Pro trap (BGP), the Centers for Disease Control miniature light trap (LT), and the Mosquito Magnet Patriot trap (MM) for mosquito surveillance in Scotland and contributes to the wider literature in low-density, temperate settings. Across two field experiments in an urban marsh in Glasgow, we found that the BGS, BGP, and LT traps performed similarly in terms of both the numbers and diversity of mosquito species caught. In contrast, the MM collected substantially more mosquitoes, potentially because of its higher and more consistent CO_2_ production rate and its greater catch of *Anopheles claviger*. Mosquito species diversity also varied by trap type, with relatively high numbers of mammalophilic *Anopheles* and *Aedes* species collected by the MM. In contrast, *Culex pipiens* s.l. (of which only the primarily ornithophilic biotype *Cx. p. pipiens* [7] was identified) was collected only by the BGS, BGP, and LT. There was no single optimal method that consistently collected more of the different mosquito species than the others; therefore, a combination of different trapping methods may be necessary to estimate the abundance and diversity of mosquito species in Scotland.

Across both experiments, mosquito abundance was relatively low and variable (1.61 per day ± 1.89), likely reducing statistical power to detect anything but large differences between trap types. Consequently, although statistically significant differences in mosquito abundance between the BGS, BGP, and LT were not detected here, moderate differences between these methods cannot be ruled out. However, our findings are similar to those reported in other settings, including the lack of significant differences between BGS and LT for relative abundance in the USA [85] and between BGP and BGS in Mozambique [59]. While the BGS and BGP were primarily developed for trapping day-biting *Aedes aegypti* and *Ae. albopictus* in domestic settings [42, 43], they have been successfully used for surveillance of a range of mosquito taxa across diverse ecological settings, including wetlands [23, 61]. We therefore do not attribute their lower performance relative to the MM to habitat unsuitability, but rather to differences among the bait types used. The MM is designed as an outdoor household trap to reduce human biting [46, 86] and thus utilises an octenol lure designed to attract mammalian biting species [87, 88], such as *Anopheles claviger*. Additionally, the MM has a higher and more consistent CO_2_ production rate than other methods (due to its reliance on a propane tank rather than a yeast mixture), which likely attracts more mosquitoes, particularly mammalophilic species [61, 89, 90]. Whilst the use of a yeast mixture to produce CO_2_ in the other trapping methods is highly cost-effective and readily available, the volume of CO_2_ produced is often variable at ambient temperature, with production declining after a few hours and potentially stalling at lower temperatures [69, 91]. Furthermore, the consistent production of CO_2_ by the MM may also make it more attractive to nocturnal and crepuscular species such as *An. claviger* [92], while CO_2_ output from yeast fermentation in the other methods is likely lowest during the cooler overnight periods when nocturnal species are most active. Our findings are broadly consistent with other studies from the UK and globally [55, 56, 60, 61], which found that the MM caught significantly more mosquitoes than any other trap, largely due to its enhanced capacity to sample *Anopheles* and *Aedes* mosquitoes.

As poikilothermic organisms, mosquito life history and behavioural traits, such as survival and host-seeking, are strongly influenced by ambient temperature [93, 94]. Across both experiments, daily mosquito abundance was predicted to rise with minimum daily temperature (over a range of 5.4–13.8 °C across experiments). This is consistent with the existence of a minimum temperature threshold below which mosquito activity cannot occur [95, 96] and with reduced mosquito flight and host-seeking behaviour observed at lower temperatures [97, 98]. Conversely, mosquitoes also have an upper thermal tolerance limit [98, 99], beyond which their fitness and activity may be disrupted. The negative association between mosquito catch rates and maximum temperatures in Experiment 2 (ranging from 14.9 to 23.5 °C) may indicate that climatic conditions were approaching the thermal maxima for these northern mosquito populations. Thermal performance and tolerance vary within and between species and populations [100–102], raising the possibility that Scottish mosquito populations have a lower thermal tolerance than those reported at lower latitudes. However, this local adaptation hypothesis cannot be separated from an alternative explanation: that the reduced abundance of mosquitoes in traps at higher temperatures may reflect reduced trap attractiveness due to temperature-driven changes in yeast-generated CO_2_ release. Given the strong temperature dependence of yeast fermentation in CO_2_ production, it remains unclear whether observed associations between mosquito abundance and temperature reflect mosquito activity or variation in CO_2_ production. To address these confounding variables, further research should be conducted on temperature and mosquito activity patterns in temperate settings such as this, using a trap method with more standardised CO_2_ release. Furthermore, more work across a broader seasonal window, which would generate larger sample sizes, is needed to determine species-specific thermal limits in Scotland, particularly under predicted climate change scenarios.

The eight mosquito species collected in this study are broadly expected to be found in reedbeds and wet woodland wetlands in Scotland and the rest of the UK [23, 24, 103]. There were some minor differences in the species collected and in the diversity estimates between the BGS, BGP, and LT, but sample sizes were too small for robust comparisons. Notably, all three traps successfully collected *Culiseta morsitans*, *Culex pipiens* s.l., and *Culiseta annulata*, all of which are potential enzootic and bridge vectors for emerging VBDs in the UK, such as WNV and USUV [12]. In contrast, in Experiment 2, the BGS caught only one mosquito, much less than the other methods. This demonstrates the context-specific nature of trap performance, with seasonality and the composition of mosquito communities likely influencing results even over relatively short sampling periods. The introduction of the MM trap in this experiment may also have influenced trap performance by potentially outcompeting the other trap types.

In addition to collecting the highest number of mosquitoes, the MM had the highest species richness of any trap type. However, species diversity in the MM, as estimated by the Gini-Simpson and Shannon indices, was relatively low. This was due to catches using this method being dominated by a single species (*An. claviger*) and to the low representation of other potential enzootic vector species (*Cs. morsitans* and *Cx. pipiens* s.l.) compared to other methods. Similarly, a study in The Netherlands found significantly more *Cx. pipiens* were collected with LTs than with MMs [57]. This difference in the number of bird-biting species collected was likely not caused by the olfactory bait type alone [104] but by the combination of MM’s use of an octenol lure, which is designed to attract mammophilic species, and its high outputs of CO_2_, which may reduce the attraction of ornithophilic species [90, 105, 106]. Conversely, lower levels of CO_2_ may be more attractive to ornithophilic species, which are adapted to respond to the lower CO_2_ release rates produced by birds [90, 105, 106]. This, in turn, may explain why these mosquitoes were captured only in traps with lower CO_2_ production rates; however, further work is needed to confirm this. Given that bird-biting species such as *Cx. pipiens* are key enzootic vectors across Europe [17], understanding how different methods perform in detecting these species under low-density conditions may be directly relevant to surveillance in other temperate regions. Additionally, these findings suggest that while the MM is effective in capturing a high number of mosquitoes and certain genera (such as *Aedes* and *Anopheles)*, it may underrepresent important ornithophilic vectors, highlighting the need for a multi-trap approach to obtain a more comprehensive understanding of mosquito community composition. Together, these findings highlight the strengths and limitations of using each trap type in the Scottish context.

Collections made with the CDC gravid trap, in parallel with host-seeking methods, here sampled 19 mosquitoes, comprising *Cs. annulata*, *Cs. morsitans*, and *Aedes cinereus*/*Ae. geminus*. Detection of these species with a gravid trap is somewhat consistent with their known larval ecology and ability to exploit artificial larval habitats. *Culiseta annulata* larvae are found in artificial containers, wetland environments, and ponds [103]. Although *Aedes cinereus/Ae. geminus* and *Cs. morsitans* are generally associated with natural aquatic habitats [103], their collection by the GTs here indicates some attraction to these traps. Although the GT used is reported as being highly effective in collecting *Culex* mosquitoes in the UK and elsewhere [107–110], none were collected despite their presence at the site. The low performance of the GT for *Culex* mosquitoes here is unclear, but it most likely reflects stronger oviposition cues (e.g. decomposing organic odours) from the nearby marsh, which created preferred oviposition sites. To address this, future studies in Scottish wetlands should consider using an oviposition medium brewed longer than 7 days to enhance its attractiveness to *Culex* mosquitoes. Lastly, although the sample size was small, 10.5% of the mosquitoes captured in the GT were visibly blood-fed at collection, 10.5% were gravid, and 79% were visually unfed, likely reflecting resting behaviour near oviposition sites [63]. This proportion is higher than that reported in a multi-year UK study conducted in zoological gardens, where ~ 5% of mosquitoes were blood-fed, albeit with substantially larger sample sizes [63]. These preliminary findings serve as proof of concept that GTs can successfully sample mosquitoes in Scotland. Unlike host-seeking traps, which are often biased toward newly emerged and unfed females, these traps are better suited for sampling mosquitoes that have previously taken at least one blood meal. Thus, gravid trap collections may be more effective for detecting pathogens in mosquitoes. Although preliminary, our results highlight the promise of this approach and justify further investigation.

While we primarily evaluated these traps on entomological efficacy, several other considerations may affect their use. Although the MM demonstrated significant value, this trap has disadvantages in terms of logistics and cost. Its reliance on a propane tank means it can only be safely used outside and out of public reach, limiting its application for wide-scale sampling in urban or public areas. The MM is also considerably more expensive than the other methods, costing upwards of £450, compared with approximately £100 to £200 for the other three methods. Lastly, the LT may also present practical issues for its use in Scotland, owing to its ability to collect large numbers of moths within reserves, which often restricts its use elsewhere in the UK [61].

This study also had some key limitations. Sampling was conducted at a single site with only two main habitat types; consequently, only species typically associated with these environments were collected. In addition, data collection was conducted over a narrow seasonal window, from the peak to near the end of the mosquito season in Scotland. These factors may have contributed to the low abundance and sample sizes in this study, limiting the generalisability of these findings to other habitat types or periods of the mosquito season in Scotland. A further key limitation was the low and variable CO_2_ production rates from yeast fermentation under temperate conditions, which may have resulted in the occasional absence of CO_2_ production during cool periods. Furthermore, while all trap types in Experiment 1 utilised this method of CO_2_ production, the inclusion of an MM in Experiment 2 limits direct comparisons between trap types because of the MM propane cylinder's much higher and more consistent CO_2_ production. The discrepancy in CO_2_ production between trap types is therefore a confounding variable that could not be controlled for in this study but should be accounted for when interpreting the findings, particularly the greater abundance of mosquitoes observed in the MM. Despite this, the data here can help inform future trapping protocols across temperate regions characterised by low mosquito densities and enhance ongoing mosquito surveillance in Scotland.

## Conclusions

Overall, our results indicate there may be no single 'best' trap for representative sampling of the mosquito population in Scotland. The trap types that consistently caught the broadest range of species across both experiments did so in relatively low numbers (for example, the BGP). In contrast, the trap type that captured the highest numbers (MM) did so primarily through high capture rates of just one to two species, whilst poorly representing ornithophilic species such as *Culex pipiens* s.l. Together, these findings indicate that effective mosquito surveillance in Scotland will require a multi-trap approach, combining highly sensitive traps such as the MM with complementary methods to increase sensitivity and ecological representation. As the risk of mosquito-borne VBDs increases in Scotland and other temperate regions worldwide, findings such as these will be essential for strengthening ongoing monitoring efforts and informing future mosquito surveillance strategies.

## Supplementary Information

Additional file1 (DOCX 2444 KB)

## Data Availability

The dataset for this study was uploaded to VecDyn via the VBD Hub, with curation support from Sarah Kelly, and is accessible upon request.

## Abbreviations

VBD: Vector-borne disease
CDC: Centers for Disease Control
BGP: The biogents BG-Pro
BGS: Biogents BG-Sentinel
LT: CDC miniature light traps
MM: Mosquito Magnet trap
GT: CDC gravid traps
USUV: Usutu virus
WNV: West Nile virus
TT mean temp: Tinytag mean temperature
TT min temp: Tinytag minimum temperature
TT max temp: Tinytag maximum temperature
TT mean RH: Tinytag mean relative humidity
WS mean temp: Weather station mean temperature
WS min temp: Weather station minimum temperature
WS max temp: Weather station maximum temperature
WS total rainfall: Weather station total daily rainfall
