# Supplementary material for: A comparative assessment of mosquito trapping methods for vector surveillance in Scotland
Source: Parasit Vectors. 2026 Jun 5;19:325. doi: 10.1186/s13071-026-07478-y (PMC13459240; doi:10.1186/s13071-026-07478-y)
Supplement: Supplementary file 1 [file 13071_2026_7478_MOESM1_ESM.docx]

**A Comparative Assessment of Mosquito Trapping Methods for Vector Surveillance in Scotland**

Meshach Lee ^1^, Georgia Kirby^1,3^, Rebecca E. Brown^1^, Alanna McCallum^1^, Jean-Philippe Parvy^2^, Susanne Krabbendam^1^, Emilie Pondeville^2^, Jolyon M. Medlock^3^, Alexander G.C. Vaux^3^, Luca Nelli^1^, Francesco Baldini^1^ & Heather M. Ferguson^1*^

**Figure S1:** Mosquito trap types used in this study**.** A) BG-Sentinel. B) BG-Pro. C) CDC miniature light trap. D) Mosquito Magnet. E) CDC gravid trap.

**Text S1**

The first PCR was performed using the primers ACEpipF, ACEtorrF, and B1246sR, as described (65), to test for *Culex torrentium or Culex pipiens* s.l. This PCR was also standardised to a 20 μl reaction volume. Each reaction contained 10 μl Taq, 0.8 μl primer ACEpipF for *Cx. pipiens* s.l, 0.8 μl primer ACEtorrF for *Cx. torrentium,* 0.8 μl primer B1246sR, 1 μl of DNA, and 6.6 μl of nuclease-free water. The PCR was performed with an initial denaturation at 95 °C for 3 min, followed by 40 cycles at 95 °C for 30 s, 55 °C for 30 s, and 72 °C for 40s, with a final 10 min extension at 72 °C. Amplified fragments were visualised on 2% agarose gels with 10 μl SYBR Safe (Invitrogen). The DNA fragment of 610 bp corresponds to *Cx. pipiens*, and the DNA fragment of 410 bp corresponds to *Cx. torrentium*.

If the sample was confirmed to be *Cx. pipiens* s.l., the CQ11 locus was then amplified to determine the biotype. This PCR was performed using the method described by (66) using the molCQ11R, pipCQ11R, and CQ11F2 primers. The assay was also standardised for a 20-µl reaction volume, and each reaction contained 10 µl of Taq, 6.6 µl of nuclease-free water, 0.8 µl of each primer, and 1 µl of the extracted DNA. This PCR then follow the same cycling conditions and visualisation protocol as the first PCR. A single 200 bp fragment corresponded to the Cx. *pipiens* biotype *pipiens*, a single 250 bp fragment would correspond to the *Cx.* *pipiens* biotype *molestus*, and if a sample presented both fragments, it would be considered a hybrid. If there was no amplification in either PCR assay, it was concluded that a problem with the sample existed, and the sample was marked as inconclusive. Positive controls for *Cx. torrentium* Cx. *pipiens* biotype *pipiens and Cx. pipiens* biotype *molestus* were acquired from colonies at the MRC-University of Glasgow Centre for Virus Research, Glasgow [Parvy *et al* 2025, in Press].

**Text S2**

Formulas for calculating the Gini-Simpson’s index and Shannon's index. 95% confidence intervals were calculated manually for the Gini-Simpson’s index using the method described in (73).

Equation 1: Gini-Simpson index

**λ= n/n-1 x ∑ ps (1-ps)** (73),

n = total mosquito abundance

ps = each species count/n,

Equation 2: Shannon's index

**H = -∑ (ni/N) log (ni/N)**(74),

N = total abundance

ni = each species count

**Table S1:** Summary of the site-level climate variables in experiments 1 and 2.

| **Experiment** | **Mean Temp (°C)**  **(SE)** | **Max**  **Temp**  **(°C)** | **Max**  **Temp range**  **(°C)** | **Min**  **Temp (°C)** | **Min**  **Temp range (°C)** | **Mean**  **Rainfall (mm)**  **(SE)** | **Total**  **Rainfall (mm)** |
| --- | --- | --- | --- | --- | --- | --- | --- |
| 1 | 14.99  (0.42) | 22.60 | 17.20 -22.60 | 6.80 | 6.80 -  13.80 | 2.89  (0.90) | 39.41 |
| 2 | 13.78  (0.53) | 23.5 | 14.90 -23.5 | 5.4 | 5.4 -  12 | 1.29  (0.47) | 15.52 |

**Table S2:** Summary of the microclimate data at each sampling station during experiments 1 and 2.

| **Experiment** | **Site** | **Mean Temp (°C)**  **(SE)** | **Max**  **Temp (°C)** | **Max**  **Temp Range**  **(°C)** | **Min**  **Temp**  **(°C)** | **Min**  **Temp Range (°C)** | **Mean**  **RH (%)**  **(SE)** |
| --- | --- | --- | --- | --- | --- | --- | --- |
| 1 | PM1 | 17.02  (0.51) | 25.85 | 18.41 - 25.85 | 6.46 | 6.46 -  18.45 | 84.22  (1.37) |
|  | PM2 | 16.78  (0.32) | 22.62 | 17.55 -  22.62 | 9.81 | 9.81 -  15.34 | 86.04  (1.18) |
|  | PM3 | 16.90  (0.46) | 22.51 | 17.80 -  22.51 | 8.88 | 8.88 -  18.90 | 82.79  (1.59) |
| 2 | PM1 | 14.47  (0.51) | 24.31 | 17.46 -  24.31 | 4.36 | 4.36 -  12.85 | 87.73  (1.33) |
|  | PM2 | 14.70  (0.44) | 23.26 | 16.74 - 23.26 | 7.54 | 7.54 -14.10 | 86.76  (1.36) |
|  | PM3 | 14.68  (0.45) | 22.61 | 15.91 - 22.61 | 6.76 | 6.76 - 13.98 | 87.27  (1.48) |
|  | PM4 | 15.64  (0.78) | 23.57 | 17.16 -  23.57 | 5.77 | 5.77 -  21.93 | 81.99  (2.42) |

**Table S3:** Chi-square (χ^2^) statistics and associated p-values for the explanatory variables retained or dropped from the nested generalised linear mixed models (GLMMs) for Experiments 1 and 2, based on likelihood ratio tests. GLMM 1 is the model that includes daily variation in site-level environmental conditions collected from the nearest Weather Station (WS). GLMM 2 instead includes daily microclimatic variation at trapping points, collected using Tinytag data loggers (TT). A p-value of 0.05 or less was considered significant.

| **Experiment Number** | **GLMM Number** | **variable** | **X^2^** | **p** |
| --- | --- | --- | --- | --- |
| 1 | 1 | Trap type | 5.183 | 0.0749 |
|  |  | **WS min temp** | **9.364** | **0.002** |
|  |  | WS max temp | 1.870 | 0.171 |
|  |  | WS total rain | 1.875 | 0.171 |
|  | 2 | Trap type | 5.7042 | 0.0577 |
|  |  | TT mean RH | 0.0157 | 0.900 |
|  |  | TT mean temp | 2.126 | 0.145 |
| 2 | 1 | **Trap type** | **162.992** | **<0.001** |
|  |  | **WS max temp** | **7.523** | **0.006** |
|  |  | **WS min temp** | **4.904** | **0.0268** |
|  |  | WS total rain | 0.646 | 0.422 |
| 2 | 2 | **Trap type** | **38.876** | **<0.001** |
|  |  | TT min temp | 2.947 | 0.860 |
|  |  | TT max temp | 0.508 | 0.476 |
|  |  | TT mean RH | 0.129 | 0.7190 |

**Table S4:** The summary statistics for both of the final GLMMs for Experiments 1 and 2. The intercept refers to the BG-pro trap (BGP). GLMM 1 is the model that includes daily variation in site-level environmental conditions collected from the nearest Weather Station (WS). GLMM 2 instead includes daily microclimatic variation at trapping points, collected using Tinytag data loggers (TT). A p-value of 0.05 or less was considered significant.

| **Experiment**  **Number** | **GLMM number** | **Variable** | **Estimate** | **Std. Error** | **z-value** | **p-value** |
| --- | --- | --- | --- | --- | --- | --- |
| **1** | 1 | WS min temp | 0.350 | 0.110 | 3.165 | **0.00155** |
| **2** | 1 | Intercept | 1.300 | 1.602 | 0.83 | 0.406 |
|  |  | BGS | -1.946 | 1.0691 | -1.82 | 0.0687 |
|  |  | LT | 0.134 | 0.518 | 0.258 | 0.796 |
|  |  | MM | 2.554 | 0.393 | 6.509 | **<0.001** |
|  |  | WS max temp | -0.223 | 0.0767 | -2.903 | **0.00369** |
|  |  | WS min temp | 0.221 | 0.0860 | 2.572 | **0.0101** |
|  | 2 | Intercept | -0.662 | 0.508 | -1.301 | 0.193 |
|  |  | BGS | -1.931 | 1.112 | -1.736 | 0.0826 |
|  |  | LT | 0.119 | 0.603 | 0.197 | 0.844 |
|  |  | MM | 2.508 | 0.489 | 5.129 | **<0.001** |

**Table S5:** Summary statistics of a pairwise comparison of each trap type in Experiment 2. GLMM 1 is the model that includes daily variation in site-level environmental conditions collected from the nearest Weather Station (WS). GLMM 2 instead includes daily microclimatic variation at trapping points, collected using Tinytag data loggers (TT). A p-value of 0.05 or less was considered significant.

| **GLMM Number** | **Contrast** | **Estimate** | **Standard**  **Error** | **z-ratio** | **p-value** |
| --- | --- | --- | --- | --- | --- |
| **1** | BGP - BGS | 1.946 | 1.070 | 1.820 | 0.263 |
|  | BGP - LT | -0.134 | 0.518 | -0.258 | 0.994 |
|  | BGP - MM | -2.554 | 0.392 | -6.509 | **<0.001** |
|  | BGS - LT | -2.079 | 1.060 | -1.961 | 0.203 |
|  | BGS - MM | -4.500 | 1.010 | -4.475 | **<0.001** |
|  | LT - MM | -2.420 | 0.369 | -6.560 | **<0.001** |
| **2** | BGP - BGS | 1.931 | 1.110 | 1.736 | 0.305 |
|  | BGP - LT | -0.119 | 0.603 | -0.197 | 0.997 |
|  | BGP - MM | -2.508 | 0.489 | -5.129 | **<0.001** |
|  | BGS - LT | -2.050 | 1.100 | -1.861 | 0.245 |
|  | BGS - MM | -4.439 | 1.050 | -4.229 | **<0.001** |
|  | LT - MM | -2.389 | 0.477 | -5.004 | **<0.001** |
